# Pollen record of the Late Pleistocene–Holocene stratigraphic sequence and current plant biodiversity from *Grotta Mora Cavorso* (Simbruini Mountains, Central Italy)

**DOI:** 10.1002/ece3.9486

**Published:** 2022-11-08

**Authors:** Alessia D'Agostino, Gabriele Di Marco, Silvia Marvelli, Marco Marchesini, Juan Manuel Martínez‐Labarga, Mario Federico Rolfo, Antonella Canini, Angelo Gismondi

**Affiliations:** ^1^ Dipartimento di Biologia Università degli Studi di Roma “Tor Vergata” Rome Italy; ^2^ Laboratorio di Palinologia e Archeobotanica C.A.A. Giorgio Nicoli Bologna Italy; ^3^ Departamento de Sistemas y Recursos Naturales, E.T.S.I. Montes, Forestal y del Medio Natural Universidad Politécnica de Madrid Madrid Spain; ^4^ Dipartimento di Storia, Patrimonio culturale, Formazione e Società Università degli Studi di Roma “Tor Vergata” Rome Italy

**Keywords:** cave, Holocene, Palaeoecology, palynology, plant biodiversity, Quaternary

## Abstract

*Grotta Mora Cavorso* (Jenne, Latium), a complex karstic system in Central Italy, has returned one of the most precious Prehistoric palaeontological and anthropological heritage. Through the analysis of pollen spectra and charcoals from cave stratigraphic levels (Late Pleistocene final phases—Holocene), the overall vegetation trend of the site was pointed out. Although taphonomy and palynology of cave deposits are complex, pollen assemblage represents a reliable source for inferring past vegetation; indeed, climatic, environmental, and cultural interactions determine fossil pollen record. Site formation processes and postdepositional bias should be generally considered in the analysis of stratigraphic sequences used to define paleoenvironmental conditions. The sediment deposits from *Grotta Mora Cavorso* showed a vegetation pattern point in out a progressive increase in woody plants from lower levels upward. Palynological investigations highlighted a changing environment predominantly characterized by cooler and perhaps more humid conditions than today, with plant subalpine and marsh communities nearby the cave. The ecological requirements of the identified plant *taxa* supplied useful indications to reconstruct ancient and modern environments of the Simbruini Mounts and the Upper Aniene River valley. This scenario, in accordance with previous faunistic and carpological findings and palynological analyses from Latium, provided a further perspective on the vegetation history, biodiversity, and climate of an important crossroads between the Adriatic and the Tyrrhenian coasts.

## Graphical abstract

A palynological study from the sediment deposits of Mora Cavorso Cave is presented. The stratigraphic layers dated back from the Late Pleistocene to the entire Holocene. Pollen analysis revealed a changing environment in the upper Aniene River Valley. An important perspective on the vegetation profile of a valuable prehistoric site was provided.

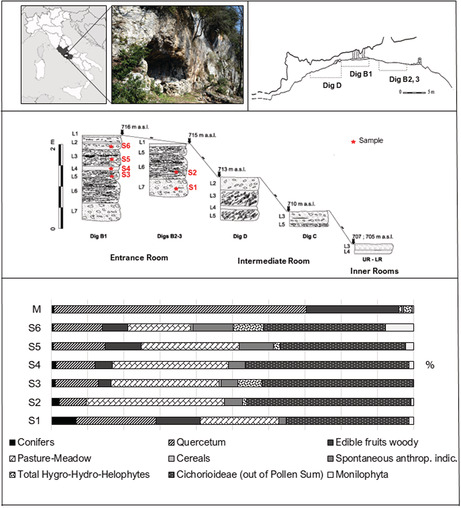

## 1 INTRODUCTION

The evolution of plants responds mainly to climatic changes. Variations in the floristic composition and structure of plant communities over time reflect past climate dynamics. The study of this phenomenon is interesting for evaluating glacial–interglacial cycles and predicting future climate modifications (Bartlein et al., 2011; Harrison & Goñi, 2010; Zhou et al., 2014).

The regions around the Mediterranean basin, during the Last Glacial period (~112 ka–11.8 ka BP), have been subjected to considerable and repeated climatic instability, which varied in agreement with local microclimate factors (Joannin et al., 2013; Sanchez‐Goñi et al., 2002; Vittori Antisari et al., 2016). In general, cooler, and drier conditions have been recorded during glacial peaks, while interglacial periods have been characterized by more humid and warm environments (Allen et al., 2000; Sanchez‐Goñi et al., 2002).

Nowadays, palynological investigations represent a precious tool to reconstruct palaeo‐phytoassociations and evaluate the response of the ancient natural landscape to human impact (Birks & Berglund, 2018). In fact, vertical stratigraphic profiles of pollen assemblages from sedimentary deposits of various origins (e.g., volcanic, marine, coastal, tectonic, and karstic) provide a continuous record of environment patterns, at centennial to multi‐millennial timescales, for different regions all over the world (Collins et al., 2012; López‐García, Blain, et al., 2014b; Louderback et al., 2011; Louderback & Rhode, 2009; Mercuri et al., 2013; Prader et al., 2020; Uzquiano et al., 2012).

The Italian Peninsula is known to be rich in Pleistocene–Holocene sedimentary deposits and several studies have been carried out to identify vegetation dynamics and climate changes occurring during the Quaternary period. In the Latium region, these palynological data have derived mainly from volcanic lakes, coastal areas, and lacustrine sites (Bellotti et al., 2011, 2016; Di Rita et al., 2010; Di Rita & Sottili, 2019; Doorenbosch & Field, 2019; Magri, 1999; Magri & Sadori, 1999; Mercuri et al., 2002; Sadori, 2018). However, in Italy, the palynological analysis of cave sediments has been rarely performed. Intriguing example is *Arene Candide Cave*, *Covoli di Velo* (Verona, Veneto), *Grotta Grande of Scario* (Salerno, Campania), *Caprelle Caves* (Macerata, Marche), and *Grotta Romanelli* (Lecce, Apulia) studied by Branch (1997), Bona et al. (2006), Ronchitelli et al. (2011), Galdenzi et al. (2014), and Ermolli et al. (2022), respectively. To date, cave palynological investigations, in Latium, are not yet published. However, several published pollen records aiming to reconstruct the landscape dynamics from Late Pleistocene and Holocene ages have been carried out in this region (Follieri et al., 1998; Galdenzi et al., 2014; Magri, 1999; Magri & Sadori, 1999; Mercuri et al., 2002; Sadori, 2018).

Caves are often valuable archeological sites, due to their content of plant, animal, and human remains. The inner stratigraphic sequences of these cavities may also provide pollen assemblages useful to reconstruct the palaeoecology of karst and montane regions (Coles et al., 1989; Hunt & Fiacconi, 2018). However, the palynology of cave deposits is a complex and challenging field of research. In fact, pollen taphonomy in caves is related to a great variety of stochastic local depositional and postdepositional events, and the models usually employed to decode pollen diagrams from open‐air sites (e.g., lakes) seem to be unsuitable (Coles et al., 1989; Hunt & Fiacconi, 2018; Turner, 1985).

As reported by Coles et al. (1989), the most important factors affecting composition and integrity of cave pollen assemblages are differential production of pollen among species, types of dispersal mechanism, energy of transport and deposition, accumulation and preservation in the cave environment, microbial decay, reworking of sediments, bioturbation, and infiltration processes.

Modern experimental approaches have provided important clues about taphonomic processes occurring on the cave pollen record and have demonstrated that the pollen rain registered inside a cave is representative of the vegetation existing in the immediate environs of the cavity (Birks & Birks, 1980; Burjachs, 1988; Camacho et al., 2000; Coles & Gilbertson, 1994; de Porras et al., 2011; Hunt & Fiacconi, 2018; Hunt & Rushworth, 2005; Loublier, 1974). Caves with simple morphology, wide entrance, and isodiametric chambers present less decay in pollen record; moreover, the anemophilous *taxa* are usually more abundant in the surroundings of the entry, while the entomophilous ones near the rear (Coles et al., 1989; Hunt & Fiacconi, 2018). Cave microclimate appears to be characterized by a diurnal pattern of airflow reversal, which allows pollen influx into the cavern (Coles et al., 1989; Cropley, 1965). Reasonably, it is possible to hypothesize that cave pollen concentration decreases with distance from the entrance, and that water input and/or animal/human intrusions make more intricate the investigations (Coles et al., 1989; Hunt & Fiacconi, 2018; Van Campo & Leroi‐Gourhan, 1956). A specific understanding of the depositional environment of sediments relative to the analyzed site must be considered, in order to facilitate the reliable interpretation of pollen presence in the cave (Coles et al., 1989).

Considering all previous evidence, *Grotta Mora Cavorso* (Simbruini Mountains, Central Italy), and the shape of the first chamber of the cave, provided the opportunity to investigate the local vegetational trend from Late Pleistocene to modern times, combining palynological analysis and floristic *census*.

**FIGURE 1:**
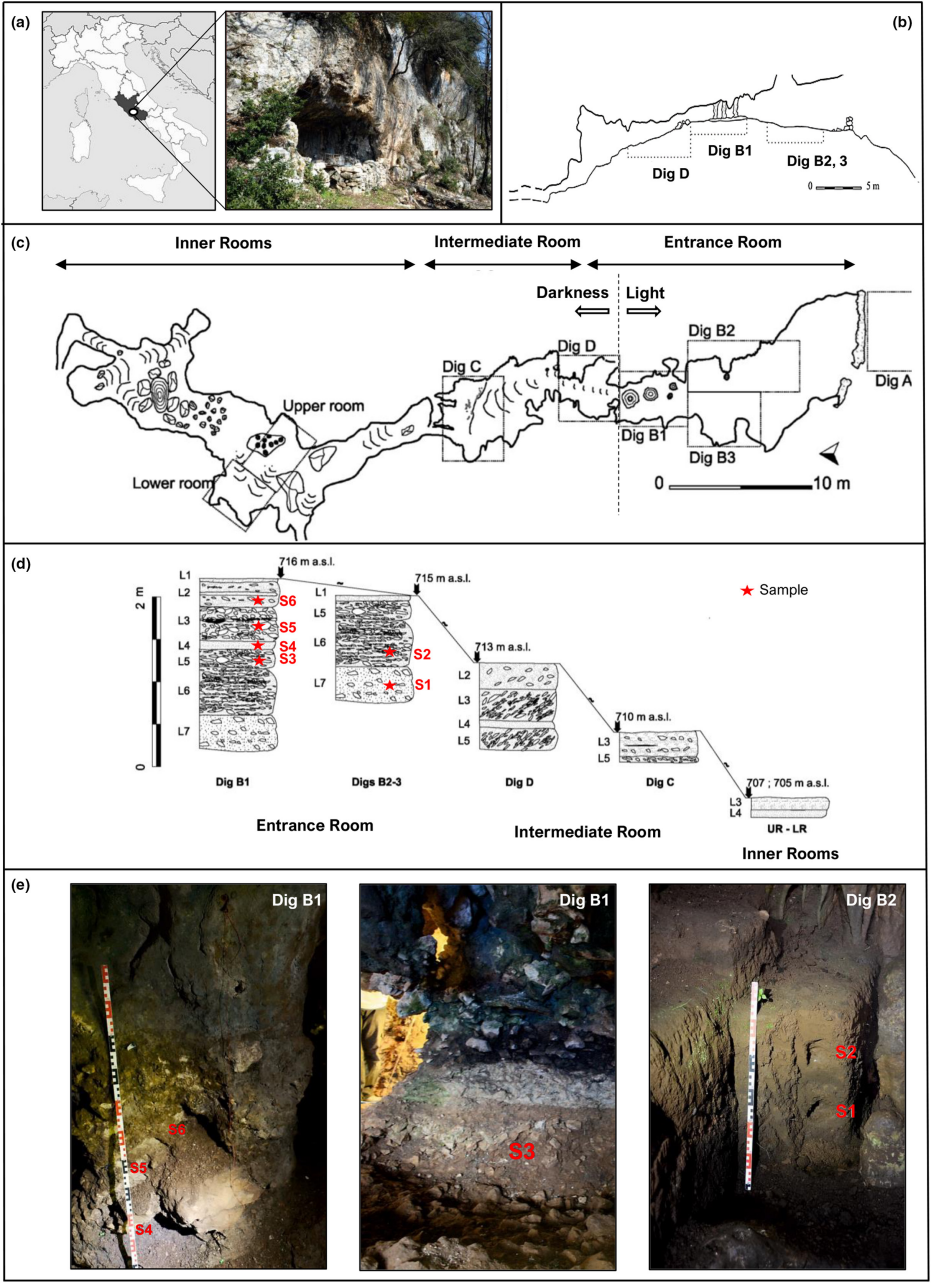
*Grotta Mora Cavorso* (Jenne, Latium, Central Italy; redrawn from Rolfo et al., 2016; Silvestri et al., 2016). (a) Map of Italy with geographical localisation of the site (white circle) and image of the cave opening; (b) transversal section of the first chamber and the first internal passage of the cave with the indication of the digs; (c) planimetry of the cave system with localization of the digs; (d) stratigraphy of the archeological digs and their height above sea level (a.s.l., in meters); sampling borings (S1–S6) were indicated by red stars (legend: L, level; LR, lower room; UR, upper room); (e) cave interior, selected digs and stratigraphical logs with the position of borin.

*Grotta Mora Cavorso*—UTM coordinates (ED50) 33T UG (03)48570(46)38010, 715 m a.s.l.—is a multi‐tunnel karst cavity located near the village of Jenne (Rome, Italy) (Figure 1). It is placed on the Eastern slope of the upper Aniene River Valley, within the Simbruini Mounts Regional Natural Park (Rolfo et al., 2016; Rolfo & Salari, 2009). The cave is part of the *Calcilutiti e Calcareniti del Conaciano‐Campaniano* formation (Upper Cretaceous) (Damiani et al., 1998; Rolfo & Salari, 2009).

In the early 2000s, the speleological group Shaka‐Zulu of Subiaco (Rome, Latium) accidentally has discovered human bones from the inner chambers of the cave, leading to systematic investigations by the University of Rome “Tor Vergata” in agreement with the Soprintendenza Archeologica del Lazio e dell'Etruria Meridionale, which are still on‐going. This rich human burial deposit of the Early Neolithic (Rolfo et al., 2016 and references therein) made *Grotta Mora Cavorso* a key site for prehistoric central Italy. The archeological excavations have identified a complex stratigraphy for this site, spanning from the Late Pleistocene up to the modern age. In the succession of chambers and passages, beyond human remains, several archeological, archaeobotanical, and palaeontological finds were found, such as pottery, rare lithic industry, wild olive and Cornelian cherry kernels, and Lateglacial micro and macro‐mammals (Gismondi et al., 2012; Rolfo et al., 2016; Salari et al., 2017; Scorrano et al., 2019).

The upper Aniene Valley appears to be an important crossroad between the Adriatic and Tyrrhenian coasts, as documented by discontinuous traces of human activity in the cave from the Late Paleolithic to historical times (Rolfo et al., 2016). In Silvestri et al. (2020), more than ten years of multidisciplinary investigation relative to the site have been collected, for obtaining a deeper report on this peculiar cave.

Therefore, the present work aims to: (i) improve the body of palaeobotanical and cultural evidence about *Grotta Mora Cavorso*; (ii) infer the floristic composition and vegetational structure of the site at specific time frames from the Late Pleistocene to the modern age; (iii) correlate previous archaeobotanical, archeological, and palaeontological data with cave pollen assemblages, assessing climatic, anthropogenic, and faunal impact on the flora; (iv) evaluate whether the general characters of the pollen spectra extrapolated from this archeological site reflect the trend in landscape dynamics observed in previous palynological studies; (v) implement the knowledge about Apennine vegetation history.

## 2 MATERIALS AND METHODS

### 2.1 Pollen sampling

Following the standardized procedures for palynological sampling, an exemplifying collection of soils, as reported in Bona et al. (2006), was performed to assess the preservation rate of the organic material in the cave context of *Grotta Mora Cavorso* and obtain information about the general characters of the vegetation. Six core borings (S1–S6; Table 1), from which sediments for pollen analysis were collected, have been performed from the stratigraphic sequences present in the entry chamber of the site (defined as *Entrance Room*). This chamber, measuring about 90 m^2^ and with an 8‐m‐wide entrance (Figure 1a–e), can be theoretically divided into two areas where the samples were taken: (i) the innermost and less exposed part (Dig B1, Figure 1b–e), which includes stratigraphic deposits dating back to the Late Paleolithic (Level 5, L5, Late MIS 2), Early Holocene (L4), Early Neolithic (L3), and Copper‐Middle Bronze Age (L2) (Figure 1d,e); (ii) the most external and illuminated part of the chamber (Dig B2–B3; Figure 1b–e), where the deposits proceed directly from the modern age to the Late Pleistocene (L6 and L7, MIS3; Figure 1d,e) (Rolfo et al., 2016). Exposed surface layers of soils were cleaned back and discarded to a depth of 10–15 cm, to eliminate potential airborne contaminants, environmental pollutants, and recent bioturbation. Details concerning the thickness of the sampled sections can be found in Figure 1d,e. Unfortunately, there was no comparable soil stratigraphy outside the cave. Samples were immediately transferred to the Laboratory of Botany (Dept. of Biology—University of Rome “Tor Vergata”) and then to the C.A.A. Giorgio Nicoli S.r.l. (San Giovanni in Persiceto, Bologna) for palynological analyses. In addition, moss cushions (M) were gathered in the environs of the cave for checking the extraction method and testing the current pollen rain. The radiocarbon dates were calibrated with the software OxCal (version 4.4.4, Bronk Ramsey, 2021), using the Int Cal20 curve (Reimer et al., 2020) (Appendix S1).

**TABLE 1:** Main descriptive elements of the investigated sediment profiles. Stratigraphic levels, sedimentological features, and radiocarbon dating details are reported.

Samples	Level	Sediments (composition)	Laboratory	Material dated (sample type)	Radiocarbon date BP	Calibrated date BCE	Chronology
S6	L2	Dark brown clay with rare clasts and some charcoals	Lyon‐5203	Charcoal	4775 ± 35	3641–3512	Copper‐Middle Bronze Age
S5	L3	Grayish‐brown clay with abundant clasts, rich in ashes and charcoal fragments	Lecce‐LTL6124A	Charcoal	6505 ± 50	5561–5365	Early Neolithic
S4	L4	Thick concretions	Beta Miami‐227131	*Cervus elaphus* tooth	8770 ± 60	8011–7601	Early Holocene
S3	L5	Silty pale reddish‐brown paleosol, with abundant decimetric clasts and several patches of carbonate crust.	LSCE–Paris‐48124	*Marmota marmota* lower jaw	14,170 ± 70	15,445–15,107	Late Upper Paleolithic (Late MIS 2)
S2	L6	Silty pinkish‐brown paleosol with abundant centimetrical clasts	LSCE–Paris‐48125	Mixed microvertebrate remains	32,770 ± 660	37,382–34,099	Late Pleistocene (MIS 3)—Unit 2
S1	L7	Silty reddish‐brown paleosol with rare clasts	LSCE–Paris‐48126	*Rupicapra rupicapra* tooth	35,000 ± 870	39,962–36,168	Late Pleistocene (MIS 3)—Unit 1

### 2.2 Pollen analysis

A standard methodology already tested for pollen substrates was applied with some minor modifications (Lowe et al., 1996; Marchesini et al., 2017). *Lycopodium* spore tablet was introduced in each sample and used as a *spike* to calculate pollen concentration (expressed as pollen grains per gram, p/g). About 10 g of sediment was deflocculated in 10% Na‐pyrophosphate. The residue was subsequently washed, filtered through nylon sieves, and resuspended in 10% hydrochloric acid, for 24/48 h, to remove calcareous material. Erdtman acetolysis was performed on the samples for 10 min; a heavy liquid separation method was applied, using sodium metatungstate hydrate at different concentrations, and then, centrifugation at 2000 rpm for 20 min was carried out, to concentrate pollen and remove extraneous organic and inorganic materials. The retained fractions were treated with 40% hydrofluoric acid, for 24 h, washed in ethanol, and desiccated at 70°C. Finally, the sediment was mounted on a glass slide by glycerol jelly and sealed with paraffin. Identification of the samples was performed at 1000 light microscope magnification (ocular ×10 and objective ×100). The determination of pollen grains was based on the *Palinoteca* of the C.A.A. Giorgio Nicoli S.r.l. laboratory (Italy), atlases, and a vast amount of specific morphopalynological literature. Names of the families, genus, and species of plants conform to the classifications of Italian Flora proposal by Pignatti et al. (2017–2019) and European Flora (Tutin et al., 1964–1980, Tutin et al., 1993). The pollen terminology was based on Berglund and Ralska‐Jasiewiczowa (1986), Faegri and Iversen (1989), and Moore et al. (1991), with slight modifications that tend to simplify the nomenclature of plants. The term *taxon* was used in a broad sense to indicate both the systematic categories and the pollen morphological types (Beug, 2004). For each sample, at least 500 pollen grains were counted, and the identified *taxa* have been expressed as percentages of the total pollen sum that includes only terrestrial pollen, without fern spores and Cichorioideae (to avoid the possible masking effects of local vegetation by the species of this family). Based on vegetational and ecological characteristics, the following main pollen groups were identified: conifers (*Pinus*), deciduous trees (this group includes Quercetum *taxa*—*Quercus*, *Carpinus betulus*, *Corylus avellana*, *Fraxinus*, *Ostrya carpinifolia*, *Tilia* and *Ulmus* + other deciduous trees), meadow (this group mainly comprises Fabaceae, Asteroideae, Caryophyllaceae, and Poaceae), and anthropogenic indicators (e.g., Cerealia, *Chenopodium*, and *Urtica*). Indeterminate grains, Monilophyta spores, *Alia* (Concentricystes), and secondary deposition granules were calculated in percentage on the pollen sum plus themselves, according to Berglund and Ralska‐Jasiewiczowa (1986). The secondary deposition grains were rare, except for sample S3, which showed the highest value of the series.

### 2.3 Anthracological analysis

Three charcoals collected from the inner chambers of the cave were subjected to anthracological analysis. In the laboratory of C.A.A. Giorgio Nicoli S.r.l., they were identified using a reflected light microscope with the help of the anthracological reference collection and on the keys and atlases (Grosser, 1977; Hather, 2000; Jacquiot et al., 1973; Schweingruber, 1990).

### 2.4 Floristic *census*

From a biogeographic point of view, the area under investigation was assigned to the Apennine‐Balkan Province and Apennine Sub‐province (Blasi & Biondi, 2017; Rivas‐Martínez et al., 2004). The detection of the current flora present in the surroundings of *Grotta Mora Cavorso* was carried out in two excursions between 2009 and 2019, along two transects. Plant species were noted and photographed, to perform their identification. The classification of the species was carried out according to the most recent taxonomic and systematic criteria, as reported by Bartolucci et al. (2018), Galasso et al. (2018), and Pignatti et al. (2017–2019) for the Italian Flora. For some genera, modern monographs were also considered, such as Peccenini and Polatschek (2016) for *Erysimum*. The GBIF portal (2020) was consulted to check the presence of the identified plants in the territory. The nomenclature of specific and subspecific *taxa* was in accordance with IPNI (2012 onwards), The Plant List (2013 onwards), Euro+Med (2006 onwards), Tropicos (2017), and Biondi et al. (2014). *Taxa* at varietal rank and hybrids were not considered.

## 3 RESULTS

**FIGURE 2:**
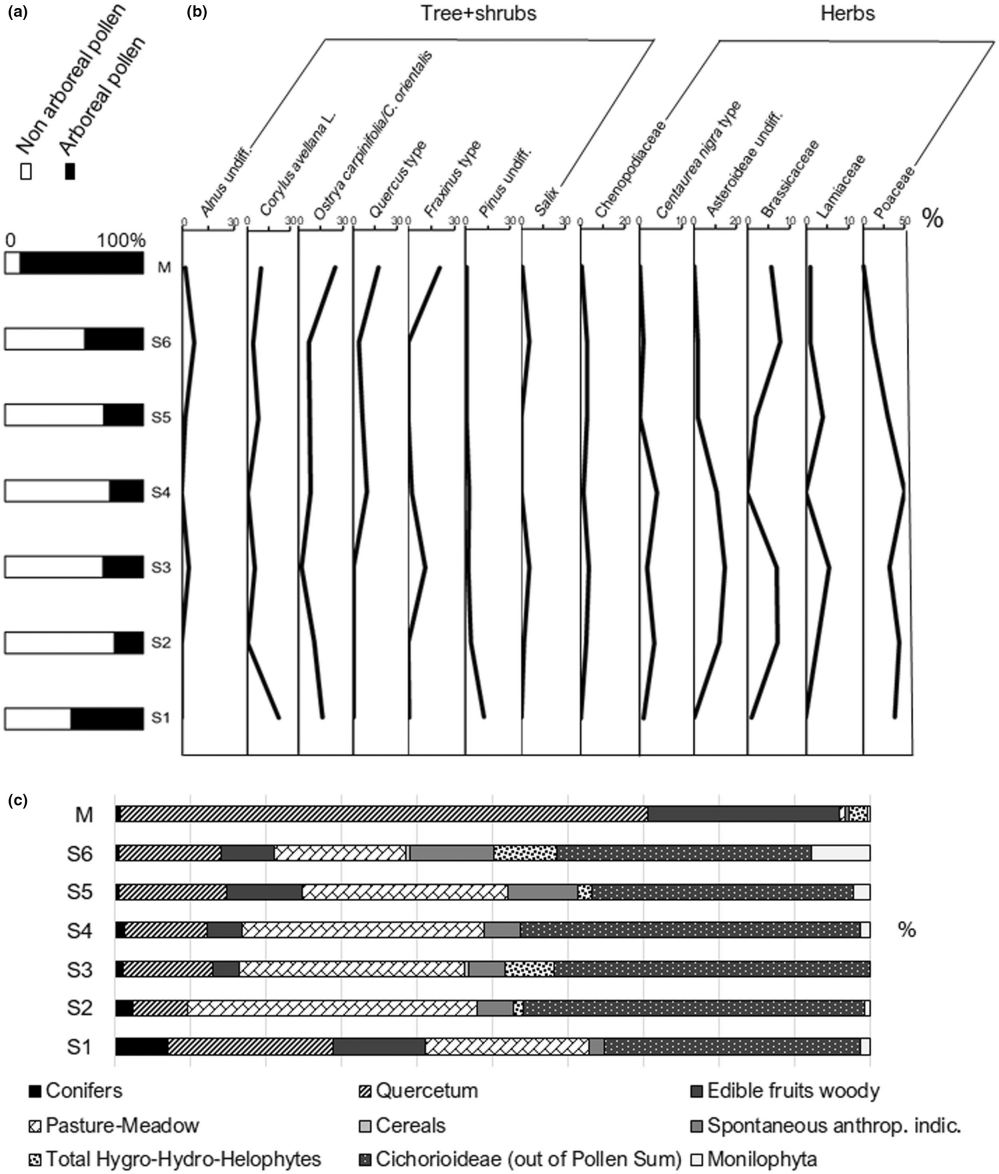
Pollen percentages obtained by palynological investigation (S1–S6 and M): (a) histograms of the arboreal pollen/nonarboreal pollen (AP/NAP) ratios; (b) diagrams of some specific *taxa*; (c) histograms of the main ecological pollen groups.

Pollen was found in all samples in a sufficiently good state of preservation, allowing its identification and testifying that sediments were conservative (e.g., pH, organic component). Overall, about 4000 pollen grains and fern spores were counted in the 6 sample depths and in the modern moss cushion. The pollen flora consisted of 56 types (i.e., 20 trees, 33 herbs, and 3 ferns) (Appendix S2). Palynological results, including changes in arboreal pollen/nonarboreal pollen (AP/NAP) ratio, were presented as pollen percentage histograms and diagrams (Figure 2). The Cichorioideae pollen is often abundant in palynological preparations, due to its resistance to degradation events, easily recognizable morphological structure (although fragmented and corroded), and the great distribution of these Asteraceae species (Galdenzi et al., 2014). For this reason, data analysis did not include this pollen type. Pollen concentration was variable, about 10^3^–10^6^ pollen per gram of sediment and 10–10^3^ Monilophyta spores per gram of sediment, depending on the richness of the organic matter and the preservation conditions. Additionally, to evaluate the floristic diversity and the human‐environment interaction, two indices were calculated: the Floristic Richness Index (FRI: number of *taxa* in one sample/number of *taxa* in all deposits × 100; Hubbard & Clapham, 1992) and the Human Influence on Flora Index (HIFI: number of *taxa* of anthropogenic indicators/total number of pollen types of the sample × 100; Accorsi et al., 1998). While the first parameter shows the floristic richness of the sample compared with the pollen diversity detected in the investigated site, the second value documents the anthropogenic influence on the vegetation regarding the chronology. The FRI showed a good floristic variability in the samples (from 26.8% to 64.3%, average 45.55%), while the HAFI documented discrete values in almost all samples (from 0.5% to 53.3%, average 26.9%) (Appendix S1).

The data of each stratigraphic horizon are described below, independently and in chronological order.

### 3.1 Sample S1: Late Pleistocene (MIS 3)—Unit 1

This sediment was collected in Dig B2 (L7) and was radiocarbon‐dated at 35,000 ± 870 year BP (39,962–36,168 cal year BCE).

The layer, corresponding to the stratigraphically lowest, was characterized by a forested landscape (A/NAP = 52.4/47.6) shaped by various deciduous Quercetum trees (*Corylus avellana* 22.2%, *Ostrya carpinifolia*/*Carpinus orientalis* 16.6%, *Fraxinus excelsior* type 0.9%), and conifers (*Pinus* 12.7%). The herbaceous *taxa* of this spectrum presented Poaceae (38.3%) and *Urtica dioica* type (2.8%), accompanied by several *taxa* (0.9% each): *Aster* type, *Centaurea nigra* type, Brassicaceae, Caryophyllaceae, and Fabaceae (Figure 2). The percentage of Monilophyta spores was 2.5%. FRI and HIFI were 26.8 and 7.0, respectively, for this level.

### 3.2 Sample S2: Late Pleistocene (MIS 3)—Unit 2

This sample was gathered in Dig B2 (L6) and was radiocarbon‐dated at 32,770 ± 660 year BP (37,382–34,099 cal year BCE).

The landscape inferred from this horizon was characterized by an abrupt decrease in afforestation rate (A/NAP = 21.2/78.8), probably due to a climatic deterioration (Figure 2). Pollen of *O. carpinifolia*/*C. orientalis* (10.6%) and *Pinus* (3.5%) appeared considerably reduced if compared to the previous level (S1). Among the species typical of damp environment, *Salix* (1.8%) was detected; the presence of *Helianthemum* (5.3%) was also interesting. Concerning nonarboreal pollen types, Poaceae (43.8%), Asteraceae, including Asteroideae (11.9%) and *C. nigra* type (3.5%), Brassicaceae (7.1%), Chenopodiaceae (2.7%), and Lamiaceae (2.7%) overcame the pollen spectrum. Additionally, a slight amount (0.9% each) of Apiaceae, Scrophulariaceae, *Galium* type, and *U. dioica* type were recorded. Finally, Monilophyta spores were 1.1%.

### 3.3 Sample S3: Late Upper Paleolithic (late MIS 2)

This soil was sampled in Dig B1 (L5) and was radiocarbon‐dated at 14,170 ± 70 year BP (15,445–15,107 cal year BCE).

With respect to the previous horizon (S2), the arboreal and herbaceous components remained quite similar (A/NAP = 29.5/70.5); among trees, *Pinus* and *O. carpinifolia*/*C. orientalis* showed a lower percentage (1.8% each) but remained always present. On the other hand, deciduous broad‐leaved trees (i.e., *Fraxinus* 11.1%, *C. avellana* 5.5%), and hygrophylous *taxa* (i.e., *Salix* 5.5%, *Alnus* 3.7%) increased. This sample was mainly characterized by the presence of Poaceae (31.3%), Asteroideae (14.7%), Brassicaceae (5.1%), Chenopodiaceae, Lamiaceae (2.8% each), *C. nigra* type (1.8%) pollen, already dominant in the cold and dry polyphyta grassland of the previous level. *Lamium amplexicaule* type (1.8%), Caryophyllaceae, Cyperaceae, *Chenopodium* cf., “*Hordeum”* group, *Lotus* type, *Stachys sylvatica* type, *Hornungia* type, and *Sinapis* type pollen (0.9% each) were also identified. No Monilophyta spore was found (Figure 2).

### 3.4 Sample S4: Early Holocene

The sample S4 was collected in Dig B1 (L4) and was previously radiocarbon‐dated at 8770 ± 60 year BP (8011–7601 cal year BCE).

In this level, the arboreal cover slightly decreased (A/NAP = 24.3/75.7), changing in composition (Figure 2). The traces of humid environment typical species disappeared, while *O. carpinifolia/C. orientalis* (8.1%) and *Pinus* (2.7%) records increased if compared to S3. In this pollen assemblage, new species appeared: *Quercus* cf. *robur* (1.4%), undiff. Deciduous oaks (8.1%), *Fraxinus ornus* L. (2.7%), and *Tilia cordata* Mill. (1.4%). The herbaceous *taxa* could be typically associated with arid polyphyta grasslands. Poaceae pollen grains (50.0%) showed the highest percentage value of the series, followed by Asteroideae (10.8%). Low amounts (ranging from 1.4% to 4.1%) of *C. nigra* type, *Bellis perennis* type *Cichorium intybus* type, Apiaceae, Chenopodiaceae, Fabaceae, and Ranunculaceae pollen have been also recorded. Spores of Monoliphyta (2.7%) reappeared in this sample.

### 3.5 Sample S5: Early Neolithic

This sediment was recovered in Dig B1 (L3) and was previously radiocarbon‐dated at 6505 ± 50 year BP (5561–5365 cal yr BCE).

**FIGURE 3:**
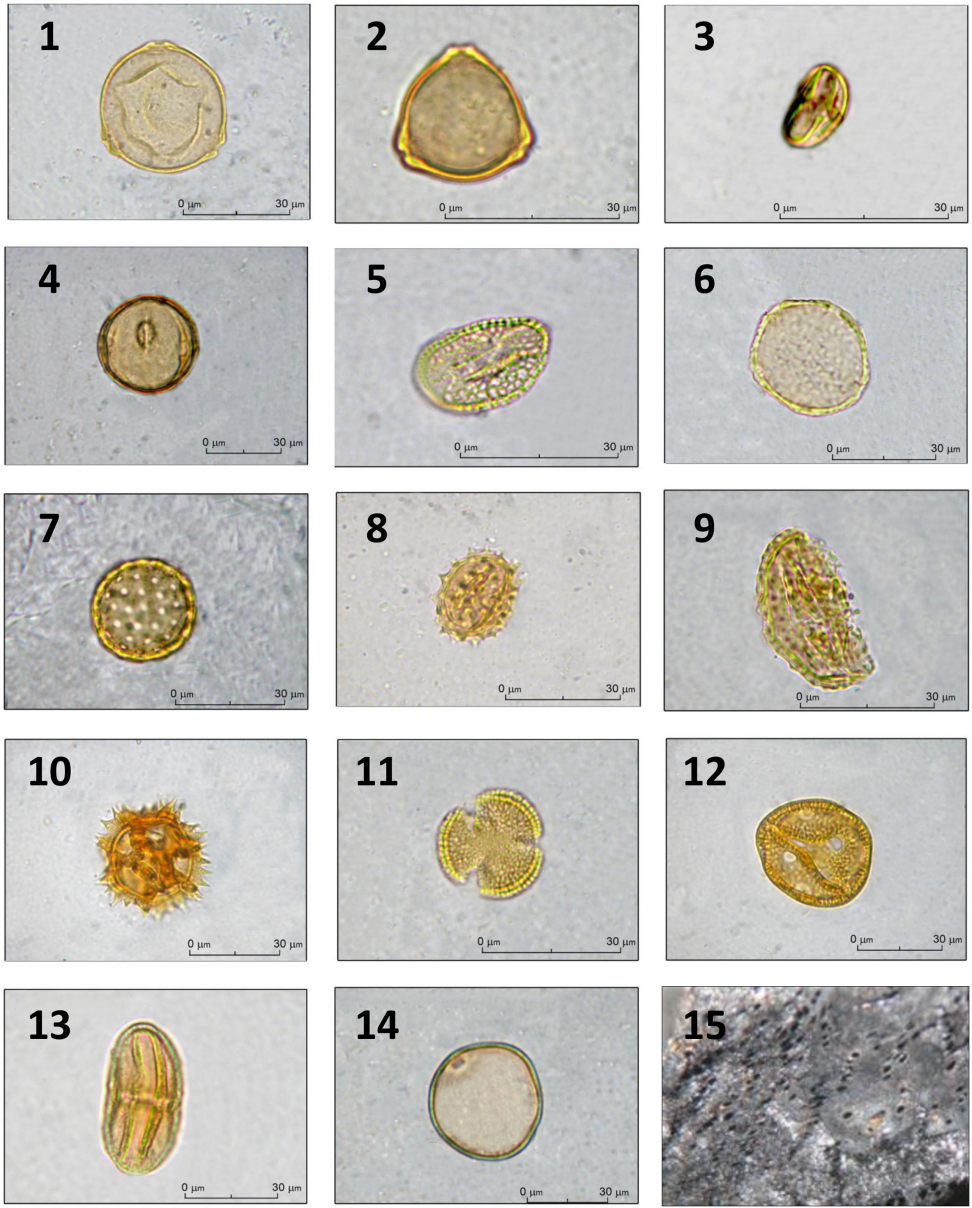
Representative images of pollen found in the stratigraphic sequence from Grotta Mora Cavorso: *Carpinus betulus* L. (panel 1), *Corylus avellana* L. (2), *Castanea sativa* Miller (3), *Fagus sylvatica* L. (4), *Salix* (5), *Ulmus* (6), *Chenopodium* cf. (7), *Aster* type (8), *Centaurea nigra* type (9), Cichorioideae undiff. (10), Brassicaceae undiff. (11), Caryophyllaceae undiff. (12), Fabaceae undiff. (13), “*Hordeum*” group (14). Anthracological sample: *Ostrya carpinifolia* (transverse section 10×) (15).

This horizon was characterized by a moderate increase in tree coverage (A/NAP = 28.6/71.4) (Figure 2). Concerning arboreal pollen types, *C. avellana* (7.6%), *O. carpinifolia*/*C. orientalis* (7.6%), and deciduous *Quercus* (5.7%) prevailed in the assemblage, followed by riparian species (*Alnus*, 1.9%) and low amounts (1.0% each) of several deciduous broad‐leaves (*Carpinus betulus*, *Ulmus*, *Castanea sativa* Mill., *Fagus sylvatica* L.), evergreen trees (*Quercus ilex* L.) and conifers (*Pinus*). It should be noticed the presence of Monilophyta (3.7%) and anthropogenic indicators, which reach a relatively high percentage (15.2%) (Figure 2). Nonarboreal pollen types were abundant in the palynological sample. The number of herbs shaping the environment increased considerably: Poaceae (29.5%), *C. intybus* type (10.5%), Ranunculaceae (3.8%), Chenopodiaceae (2.9%), Rosaceae (2.9%), *Aster* type, Asteroideae, *Lotus* type, Fabaceae, *Salvia* type and *Ranunculus acris* type (1.9% each), and *Symphytum officinale* type, *Hornungia* type, Brassicaceae, *Lamium amplexicaule* type, Lamiaceae, *Polygonum aviculare* group, and *Typha angustifolia* L. (1.0% each). In the stratigraphic sequence, only this level showed traces of Concentricystes, microorganisms that usually live in freshwater and natural wetlands (Tang et al., 2013). An anthracological analysis was applied to fragments of charcoals (dated to Neolithic period and present in the inner chambers of the cave), to outline the exploitation of natural resources by the Neolithic community of *Grotta Mora Cavorso*. Unfortunately, only one ancient sample was taxonomically recognized as *O. carpinifolia* (Figure 3), whose presence was documented in all investigated levels.

### 3.6 Sample S6: Copper‐Middle Bronze age

Sample S6 was collected in Dig B1 (L2) and was previously dated at 4775 ± 35 year BP (3641–3512 cal year BCE).

In this last stratigraphic horizon, the arboreal component increased (A/NAP = 42.4/57.6), due to the enrichment in hygrophytes (*Alnus*, 6.9%; *Salix*, 5.9%) (Figure 2). Among the trees, the presence of *C. betulus* (3.9%) *Ulmus* and *C. sativa* (2.0% each) remained low but increased if compared to the previous level (S5), while deciduous broadleaved *taxa* (*O. carpinifolia*/*C. orientalis*, 6.9%; *C. avellana*, 3.9%; deciduous *Quercus*, 3.9%; *F. sylvatica*, 1.0%) decreased. Conifers (*Pinus*, 1.0%) remained stable. New shrub‐like (*Ephedra fragilis* type, 3.0%; *Rhamnus* type, 1.0%), and arboreal species (*C. mas*, 1.0%) were found in the palynological sample. Concerning herbaceous *taxa*, Asteraceae (*C. intybus* type 12.8%; *Aster* type, 3.9%), Brassicaceae (6.9%), Poaceae (11.3%), Chenopodiaceae (3.0%) and *Lotus* type (3.0%), Asteroideae (2.0%) and Fabaceae (2.0%) prevailed. On the other hand, *R. acris* type, Ranunculaceae, and Rosaceae (1.0% each) reduced their record in the pollen spectrum. Low percentage (1.0%) of *Hornungia* type, Lamiaceae, Cyperaceae, *Urtica* type, “*Hordeum*” group, Apiaceae, and *C. nigra* type were detected. In addition, *Hedysarum* cf. (1.0%) pollen was also registered. Monilophyta spores were found (*Thelypteris*, 7.9%; Ficales 5.1%) and the value of the anthropogenic indicators remained relevant in this phase (18.7%). FRI and HIFI were the highest of the series (S5: 57.1 and 53.3; S6: 64.3 and 44.2).

### 3.7 Modern‐day vegetation cover

The floristic composition of the vegetation growing in the surroundings of *Grotta Mora Cavorso* was studied by both palynological analyses of moss cushions (M) and systematics investigations. The obtained information opens a window into the Apennine vegetational profile, estimates the relative current plant biodiversity rate, and provides clues about the climatic conditions near the site compared to the past.

Pollen analysis highlighted a rich list of arboreal *taxa* (meso‐hygrophilous forest) (FRI 46.4). The tree coverage constitutes 89.1% of the pollen spectrum. In detail, *O. carpinifolia*/*C. orientalis* (24.8%), followed by deciduous *Quercus* (10.4%), *C. betulus* (10.4%), *C. avellana* (9.9%), *Fraxinus* (9.6%), *F. excelsior* type (7.1%), *Q. ilex* type (4.3%), and *F. ornus* (3.8%), dominate the environment. *Q*. cf. *robur* (2.8%) and *Alnus* (1.9%) are also represented. Low percentages of *Rhamnus* type (0.5%), *Pinus*, *Salix*, *C. sativa*, and *C. mas* can be detected (0.9% each). Nonarboreal pollen types are less abundant in the palynological sample (10.9%); open plant association and numerous anthropogenic *taxa*, such as Brassicaceae (2.4%), *Hornungia* type (1.9%), and *Sinapis* type (1.4%), are recognizable. Slight amount of Apiaceae, Lamiaceae, Poaceae, *R. acris* type (0.9% each), Chenopodiaceae, and Ranunculaceae (0.5% each) are also identifiable. Finally, the herbaceous anthropogenic indicators appeared less pronounced in this sample (0.5%), as well as the HIFI value (0.5). The results obtained by the floristic *census* of the two transects appeared very similar and showed a forest with a high level of maturity (Ruiz de la Torre, 1990), which remains quite stable. The complete list of the flora is shown in Appendix S3 and arranged in alphabetical order of families. A total of 135 species were identified and grouped into 41 families and 109 genera.

The dense mixed deciduous forest is dominated by *Q. pubescens* (patronymics in Appendix S3), *Acer monspessulanum*, *F. ornus*, and *O. carpinifolia*, but *Q. ilex* subsp. *ilex*, *A. opalus* subsp*. obtusatum*, and *Ficus carica* are also found. The nemoral environment is confirmed by the presence of typical forest species, some of them with a lianoid *habitus*, such as *Dioscorea communis*, *Clematis vitalba*, *Aristolochia lutea*, *Helleborus foetidus*, *Ruscus aculeatus*, and *Asparagus acutofolius*. This type of forest is very frequent in the Apennine Sub‐province (Blasi & Biondi, 2017). From the phytosociological point of view, it is located at the upper limit of the holm‐oak grove of the alliance *Fraxino orni‐Quercion ilicis* Biondi, Casavecchia & Gigante ex Biondi, Casavecchia & Gigante in Biondi, Allegrezza, Casavecchia, Galdenzi, Gigante & Pesaresi 2013, and with a few elements of *Q. pubescens* woods, which approximate the oak groves of the order *Quercetalia pubescenti‐petraeae* Klika 1933 (Biondi et al., 2014).

Among bushes, *C. sangunea*, *Pistacia terebinthus*, *Lonicera etrusca*, *Crataegus monogyna*, *Prunus mahaleb*, *Rosa* sp., and *Rubus ulmifolius* were found. The latter was typical of the class *Rhamno catharticae‐Prunetea spinosae* Rivas Goday and Borja ex Tüxen 1962, in which shrubland and mantle communities are related to the deciduous forests of the *Querco‐Fagetea* class (Biondi et al., 2014). Among retamoid legumes, *Spartium junceum*, *Colutea arborescens*, *Genista monspessulana*, and *Cytisus villosus* are identified. Some of them can be framed in a specific way in the order *Cytiso villosi‐Telinetalia monspessulanae* Rivas‐Martínez, Galán & Cantó in Rivas‐Martínez, T.E. Díaz, Fernández‐González, Izco, Loidi, Lousâ & Penas 2002 (Biondi et al., 2014).

Ferns, such as *Asplenium trichomanes* subsp. *quadrivalens* and *A. ceterach*, were found among the rocks and, together with *Parietaria judaica*, *Sedum sexangulare*, and *S. album*, testify the existence of rocky casmophytic communities in the landscape of Mora Cavorso. They can be classified within the classes *Asplenietea trichomanis* (Br.‐Bl. in Meier & Br.‐Bl. 1934) Oberdorfer 1977 and *Parietarietea judaicae* Oberdorfer 1977 (Biondi et al., 2014).

In the herbaceous stratum of the forest, nitrophilic species, such as *Chelidonium majus*, *Geranium purpureum*, *Teucrium chamaedrys*, *U. dioica*, *Melittis melissophyllum*, and *S. sylvatica*, were documented and ascribed to the class *Galio aparines‐Urticetea dioicae* Passarge ex Kopecký 1969, typical of mesophilous to hygrophilous environments with a certain anthropogenic influence. These species can also be included in the class *Cardaminetea hirsutae* Géhu 1999, and specifically in the order *Geranio purpurei‐Cardaminetalia hirsutae* Brullo in Brullo & Marcenò 1985.

Finally, other herbaceous *taxa* typical of meadows are present on the edges of the roads. They share species of wide European distribution, such as *Plantago major*, *Anacamptys pyramidalis*, *Trifolium repens*, *Eupatorium cannabinum*, and *S. sylvatica*. These plants can be clustered in the classes *Filipendulo ulmariae‐Convolvuletea sepium* Géhu & Géhu‐Franck 1987, dominated by perennial megaforbs of humid mountainous areas in Mediterranean and Temperate macrobioclimates, and *Molinio‐Arrhenatheretea* Tüxen 1937, composed of hygrophilous and mesophilous grassland (Biondi et al., 2014).

## 4 DISCUSSION

Climatic‐vegetation trend and possible land use over time in the environs of Grotta Mora Cavorso (Central Italy) were reconstructed by pollen analyses at specific time frames. In addition, a floristic *census* was conducted to study the current vegetation around the archeological site and provide information about the Apennine landscape. Even though caution must be required due to the limited number of samples, this study provided an opportunity to examine cave sediments and issues concerning the physiographic evolution of the landscape near the investigated site from the Late Pleistocene final phases to the entire Holocene.

In this contribution, the detection of pollen from entomophilous species (above all herbaceous) is certainly due to the frequentation of animals (e.g., insects, rodents, flock), in addition to the eolian influx. Pollen of anemophilous species, however, also reached the innermost part of the cave, as well as that of the entomophilous species. Probably, it is due to the structure of the first chamber of *Mora Cavorso* cave that facilitates good air circulation for the presence of a single wide entrance. However, the complex site formation processes, cave internal topography, airflow and microclimate, vegetation physiognomy, pollination type, and animal and human behavior (e.g., biotic transport) remain the major and variable factors determining cave pollen assemblages (de Porras et al., 2011). These elements should be further investigated to better reconstruct the past environment.

Pollen spectra from S1 (Late Pleistocene, MIS 3—Unit 1) revealed the presence of various herbaceous *taxa*, which well‐grow in steppe‐type environments, associated with conifers, a low percentage of Monilophyta spores, and the total absence of swamp forest indicators, testifying a possible dry and cool climatic phase with a woodland coverage and arid grasslands, most probably correlated to a temperate oscillation of Marine Isotope Stage 3 (MIS 3). Floristic richness and human influence on flora indices showed the lowest values of the investigated series, suggesting a sporadic frequentation of the cave. As reported in the literature, this period (~60 ka–30 ka BP) was dominated by the alternation between forest development and expansion of arid zones (Fletcher & Goñi, 2008; López‐García, Blain, et al., 2014), a feature prevalently observed in Mediterranean Europe (Allen et al., 2000; Sanchez‐Goñi et al., 2002). More specifically, the climate of the Latium region was very complex, due to its geographic position and variable geomorphology. Altogether, these factors produced, during the Last Glacial Period, a great variety of local climatic‐ecological patterns (Chiarini et al., 2007; Follieri et al., 1998; Magri, 1999; Magri & Sadori, 1999). The faunal assemblage of this level from *Mora Cavorso*, mainly made up of forest and arboreal species, suggested a temperate climate, colder with respect to the modern atmospheric conditions and characterized by the presence of grasslands, as reported in Salari et al. (2014) and Rolfo et al. (2016). This evidence appears in agreement with our palynological data.

The presence of *Helianthemum*, a pioneer *taxon*, could suggest dry and lightly grazed grasslands of the supra‐Mediterranean and montane vegetation zone (Buttler & de Beaulieu, 2006) in correspondence with S2 (Late Pleistocene, MIS 3—Unit 2). In addition, the presence of herbaceous and anemophilous *taxa* (e.g., Poaceae, Asteroideae, Chenopodiaceae), associated with rock rose, indicated xeric vegetation and shrub steppe formations. This remarkable development of polyphyta grasslands would prove the onset of a severe cooling in the area surrounding the cave of *Mora Cavorso*, as also supported by the lower percentage of Monilophyta spores observed in the preceding sample (S1), which, probably, introduced Marine Isotope Stage 2 (MIS 2). Similarly, the pollen spectra of *Lago della Costa* (Kaltenrieder et al., 2009) and *Valle di Castiglione* (Follieri et al., 1986) have suggested the prevalence of cold and steppic environments during this period. Animal records (i.e., microvertebrates and cold mammals) detected in this stratigraphic layer at the site allowed to correlate this level with a cold and arid climatic phase (Rolfo et al., 2016; Salari et al., 2014, 2015). This hypothesis is coherent with the present pollen‐based reconstruction.

In the Late Upper Paleolithic spectrum (S3), the high percentage of Poaceae, Asteroideae, Brassicaceae, Lamiaceae, Chenopodiaceae, and Caryophyllaceae might indicate a drier steppe biome and grazed sites; on the other hand, hygrophytes (i.e., *Salix*, *Alnus*, and Cyperaceae) could potentially mark a period of available moisture and, consequently, a wetter environment. Although the steppe *taxa* remained rather constant, a few wet‐needing plants could reflect a cool forest–steppe system with progressive climatic amelioration after the Last Glacial Maximum, probably in the phase that preceded the Bølling‐Allerød interstadial (Ravazzi et al., 2007). Indeed, between 22,000 and 14,000 year BP, a very little closed woody vegetation has been recorded in the Mediterranean area from about 500 m above the current sea level (Di Rita et al., 2013; Tzedakis & Bennett, 1995; Vacchiano et al., 2017). Lithic artifacts, typical of the final Epigravettian, together with numerous vertebrate fossils presenting butchery signs (Salari et al., 2011), represent the earliest evidence of the human frequentation to *Grotta Mora Cavorso*, probably when the inner Apennines were more accessible (Rolfo et al., 2016). In S3 sample, pastureland and cereal markers, and anthropogenic indicators, as well as pollen of *Sinapis* type, *Hornungia* type, and *Lotus* type, could be associated with the previous archeological and paleontological findings, although drought‐tolerant xeric vegetation does not necessarily imply human impact (Collins et al., 2012).

The pollen diagram obtained from S4 (early Holocene) showed nearly treeless vegetation (Figure 2). However, the detection of new temperate broad‐leaved deciduous forest *taxa* could indicate a possible transition from steppe condition to reforestation, characterized by the growth of woodlands typical of the early postglacial interval (10–7 ka BP) (Pini, 2002). Indeed, the Late Glacial (14.7–11.7 ka BP), which precedes the Holocene, was generally marked out by pronounced climatic oscillations, namely loss of forests and re‐integration of cool steppe biomes, alternating with warm and humid environments (Ravazzi et al., 2007). In addition, the Holocene is an epoch with more stable temperature (continental climate) and the presence of forests, which has been subdivided into four periods, with different climates and vegetation: Preboreal (11.7–9 ka BP), Boreal (9–8 ka BP), Atlantic (8–5 ka BP) and Subboreal (5–2.5 ka BP) (López‐García, Berto, et al., 2014; Sadori, 2018). In southern Europe, and especially in Italy, a unique pattern of vegetation is still difficult to establish, due to its high physiographic variability and land use (Di Rita et al., 2018; Magri et al., 2015, 2017). Therefore, the studied horizon could be collocated at the Boreal/Atlantic transition, and the landscape outlined by palynological analysis testified to wide grasslands but also open mixed‐oak forest (e.g., deciduous *Quercus* type and *Tilia* type). This phenomenon was plausibly ascribable to the establishment of more temperate conditions, although still relatively cold. In this context, the woodland expansion has also been proposed by palaeontological findings (Salari et al., 2015).

Pollen analysis from sample 5 (early Neolithic) suggested the existence of woodland patches alternated with temperate‐alpine meadows and steppes, in the surroundings of the cave. The forests on the hilly slopes might be hypothesized to include *C. avellana*, deciduous oaks, *C. betulus*, and *Ulmus* sp.; in particular, in well‐drained soils, *O. carpinifolia/C. orientalis* and Mediterranean evergreen elements (e.g., *Q. ilex*) also could grow. Among the mountain *taxa*, *C. sativa* and *F. sylvatica* were registered. Pteridophyte (which usually grow in the brushwood with a significant rate of humidity), Concentricystes, traces of *T. angustifolia*, *R. acris*, and swamp forest indicators (e.g., *Alnus* sp.) could testify a humid environment and/or a zone with freshwater influence. Compared to the previous level (S4), herbaceous vegetation was more differentiated but always dominated by Poaceae spontaneous species, which mainly grow in open plant associations and dry environments. From a climatic point of view, the increase in deciduous forests, compared to S4, might indicate a shift toward higher temperatures and an environment still quite arid with limited rainfalls. This reconstruction is coherent with all Holocene palynological records from Latium, which has shown a vegetation generally characterized by mixed‐oak forests, associated with *C. betulus*, *O. carpinifolia*/*C. orientalis*, *Q. ilex*, *Fagus*, and *Alnus* sp. (Bellotti et al., 2016; Di Rita et al., 2010, 2018; Magri & Sadori, 1999; Mercuri et al., 2002; Peyron et al., 2011), taking always into consideration that a linear correlation between pollen percentages registered from lake core/river sediments and cave samples cannot be expected at all. Moreover, as documented by Marchesini et al. (2020), pollen spectra from Neolithic high‐elevation sites, probably devoted to housing, have shown similar arboreal pollen percentage to that counted in our sample S5. In the Early Neolithic stratigraphic depth of *Grotta Mora Cavorso*, *Cornus mas* L. and *Olea europaea* L. seeds have been found and genetically identified (Gismondi et al., 2012), although they were not registered during the pollen analysis of this layer. This absence could be also justified by the fact that high‐altitude sites rarely have shown pollen of olive (Reille, 1992) and that the complex cave depositional dynamics had reduced the probability to detect *C. mas* traces; in fact, cornelian cherry pollen was recorded in the next layer (S6 sample). In addition, the presence of *O. europaea* seeds in the chambers of the cave could be attributable to fortuitous anthropogenic input. This species could also have been gathered far from the cave and used as firewood, as evidenced by numerous charcoals and ash found in the internal chambers. In the Neolithic layer, the anthropogenic indicators reached a high percentage. In fact, for this period, various human and faunal remains, traces of hearths, and pottery have been found, making it possible to hypothesize both domestic and burial/ritual uses for the cave chambers (Rolfo et al., 2016). Thus, for this horizon, the palynological reconstruction of the plant biome surrounding the cave appeared in line with the landscape outlined by the analysis of the micro and macro‐mammal remains (Salari et al., 2014, 2015).

In the last spectrum (S6), concerning Copper‐Middle Bronze Age, and in the previous layer, the floristic richness and human influence on flora indices were the highest of the series, maybe due to an increase in the frequentation by humans and animals. In this period, the composition of evergreen‐deciduous temperate forests did not show significant changes with respect to that of the Early Neolithic, including species like *Quercus*, *Corylus*, *E. fragilis* Desf., *Rhamnus*, and *Hedysarum*. The woody vegetation was accompanied by an intensification of species typical of damp environment and Monilophyta, reflecting a higher humidity condition, probably linked to an increment of rainfalls, an increase in temperature rate, and/or the nearness to the Aniene River. The herbaceous vegetation was always dominated by a discreet floristic list that included numerous species peculiar to polyphyta meadows/pastures (e.g., Asteraceae, Poaceae). In this context, traces of pollen grains referable to the “*Hordeum*” group, which includes cultivated barley, einkorn wheat, and various wild species, might indicate the presence of cereals (Figure 2). The anthropogenic indicators remained relevant in this phase, reaching the highest percentage of the soil sediments. The Copper‐Middle Bronze Age deposits of *Mora Cavorso* included almost four disturbed human burials, ceramic fragments, an upside‐down bowl, and numerous perinatal faunal remains, indicating a strong ritual value of the cave in that period. Based on paleontological studies, the paleoenvironment has shown a contraction of steppe areas in favor of forests, in the upper Aniene valley (Salari et al., 2014, 2015), as corroborated by our palynological analysis.

About the modern‐day vegetation, pollen analysis of the moss cushion highlighted a rich list of arboreal *taxa*. The current plant landscape changes radically compared to the ancient samples, and the environs of the cave appear immersed in a meso‐hygrophilous forest. Nonarboreal pollen types are less abundant and open plant associations and numerous anthropogenic *taxa* were identified. Finally, the herbaceous anthropogenic indicators and the human influence on the flora index appeared less pronounced in this sample. Indeed, nowadays, the cave is secluded and enveloped in a thick forest. The floristic *census* amplified the palynological evidence about the species growing on the hilly slopes surrounding the studied cave. In particular, the systematics approach enriched the knowledge about the undergrowth plant diversity. The vegetation of the area is dominated by deciduous and sclerophyllous species, a type of forest very frequent in the Apennine Sub‐province (Blasi & Biondi, 2017), adapted to mild summers and cold winters. In addition, the occurrence of a forest made up of typically different vegetation levels is common in limestone fluvial canyons (Ruiz de la Torre, 1990). The presence of acidophilic species demonstrates the high rainfall recorded in *Mora Cavorso* site, which allows limestone to be quite free and determines an acidic soil promoting the growth of calcifugal floristic elements. The hygrophilous and mesophilous grassland is characterized by ephemeral therophytic sciaphilous nitrophilous small vegetation, representative of natural and seminatural habitats (Biondi et al., 2014).

## 5 CONCLUDING REMARKS

In this contribution, we investigated the pollen‐vegetation trend of *Grotta Mora Cavorso*, a key archeological site for the Italian prehistory, by the analysis of sediment samples, promoting the value of palaeobotanical analyses in such type of depositional environment. It would be interesting to extend the same type of analysis to sediments of other nearby caves, to make a comparison with the pollen spectra obtained in this study. However, discrepancies in the sediment deposition and local climatic conditions may complicate the hypothesized correlations, even at a regional level. Consequently, if comparisons are made at a wider scale, the difficulties increase proportionally and much caution must be applied (Follieri et al., 1998).

According to palynological results, starting from the temperate oscillation of MIS 3, the vegetation surrounding the cave was characterized by a temperate‐alpine steppe alternated with woodland patches. The primary grasslands, above the treeline, were dominated by several grassy plants. Open forests of broad‐leaved woods and pines were located presumably to lower altitudes; in fact, the dry and cold climate could have led to a forest regression. The landscape would look typical of mountain regions with hygrophytes, which could account for the presence of marshy areas. This cool forest–steppe system could reflect progressive climatic amelioration after the Last Glacial Maximum. Indeed, the Late Glacial, which precedes the Holocene, was generally marked out by pronounced climatic oscillations, namely loss of forests and re‐integration of cool steppe biomes alternating with warm and humid environments.

Starting from the Early Holocene, the pollen percentage spectra of *Grotta Mora Cavorso* would testify to an increase in evergreen‐deciduous temperate woods, dominated by oaks and including ash, linden, and elm. Simultaneously, a decrease in conifer and grass pollen suggested a higher rate of humidity (i.e., warmer conditions), also testified by the sensitive growth of wet‐needing plants and fern spores, usually associated with damp grounds. During the Neolithic and Chalcolithic periods, the paleoenvironment showed a contraction of steppe areas in favor of forests, in the Upper Aniene valley. The anthropogenic indicators reached the highest percentage of the series with various human, botanical and faunal remains, traces of hearths, and pottery recovered, making it possible to hypothesize both domestic and burial/ritual uses for the cave. In the last c.a. 4000 years, the climate would seem to be rather stable and similar to the warm conditions that prevail today. Forests dominate the vegetation of most of the Apennines, and the present‐day vegetation around *Grotta Mora Cavorso* appears constituted by a rich meso‐hygrophilous forest.

In conclusion, a botanical overview of the vegetation profile of one of the most precious Italian prehistoric sites were outlined in the current research, providing valuable information on plant biodiversity, potential land use, and climatic conditions from the Late Pleistocene to date.

## AUTHOR CONTRIBUTIONS

**Alessia D'Agostino:** Conceptualization (equal); data curation (lead); formal analysis (lead); investigation (equal); writing – original draft (equal); writing – review and editing (lead). **Gabriele Di Marco:** Formal analysis (equal); investigation (equal); writing – original draft (equal); writing – review and editing (equal). **Silvia Marvelli:** Formal analysis (equal); investigation (equal); writing – review and editing (equal). **Marco Marchesini:** Formal analysis (equal); investigation (equal); writing – review and editing (supporting). **Juan Manuel Martínez‐Labarga:** Formal analysis (equal); resources (equal); writing – review and editing (supporting). **Mario Federico Rolfo:** Conceptualization (supporting); resources (equal); writing – review and editing (supporting). **Antonella Canini:** Resources (equal); validation (supporting); writing – review and editing (supporting). **Angelo Gismondi:** Conceptualization (lead); investigation (equal); project administration (lead); resources (equal); supervision (lead); writing – original draft (lead); writing – review and editing (lead).

## FUNDING INFORMATION

This research was funded by MIUR (*Ministero dell'Istruzione*, *dell'Università e della Ricerca*—Italy) through the Grant for Fundamental Research Activity (FFABR 2017/2018) (CUP: E81I18000830005, Grant Recipient: Prof. Angelo Gismondi).

## CONFLICT OF INTEREST

The authors declare that they have no conflict of interest.

## Supporting information


Appendix S1

Appendix S2

Appendix S3

## Data Availability

Data available in article supplementary materials (Appendices [Link], [Link]).
