# Supplementary material for: Pollen record of the Late Pleistocene–Holocene stratigraphic sequence and current plant biodiversity from Grotta Mora Cavorso (Simbruini Mountains, Central Italy)
Source: Ecol Evol. 2022 Nov 8;12(11):e9486. doi: 10.1002/ece3.9486 (PMC9643123; doi:10.1002/ece3.9486)

Pollen record of the Late Pleistocene-Holocene stratigraphic sequence and current plant biodiversity from Grotta Mora Cavorso (Simbruini mountains, Central Italy)

Alessia D'Agostino, Gabriele Di Marco, Silvia Marvelli, Marco Marchesini, Juan Manuel Martínez Labarga, Mario Federico Rolfo, Antonella Canini, Angelo Gismondi

Supplementary Material 1. Radiocarbon calibration obtained by OxCal (version 4.4.4—Bronk Ramsey, 2021), using the IntCal20 curve (Reimer et al., 2020), for each sample (Cavorso S1-S6).

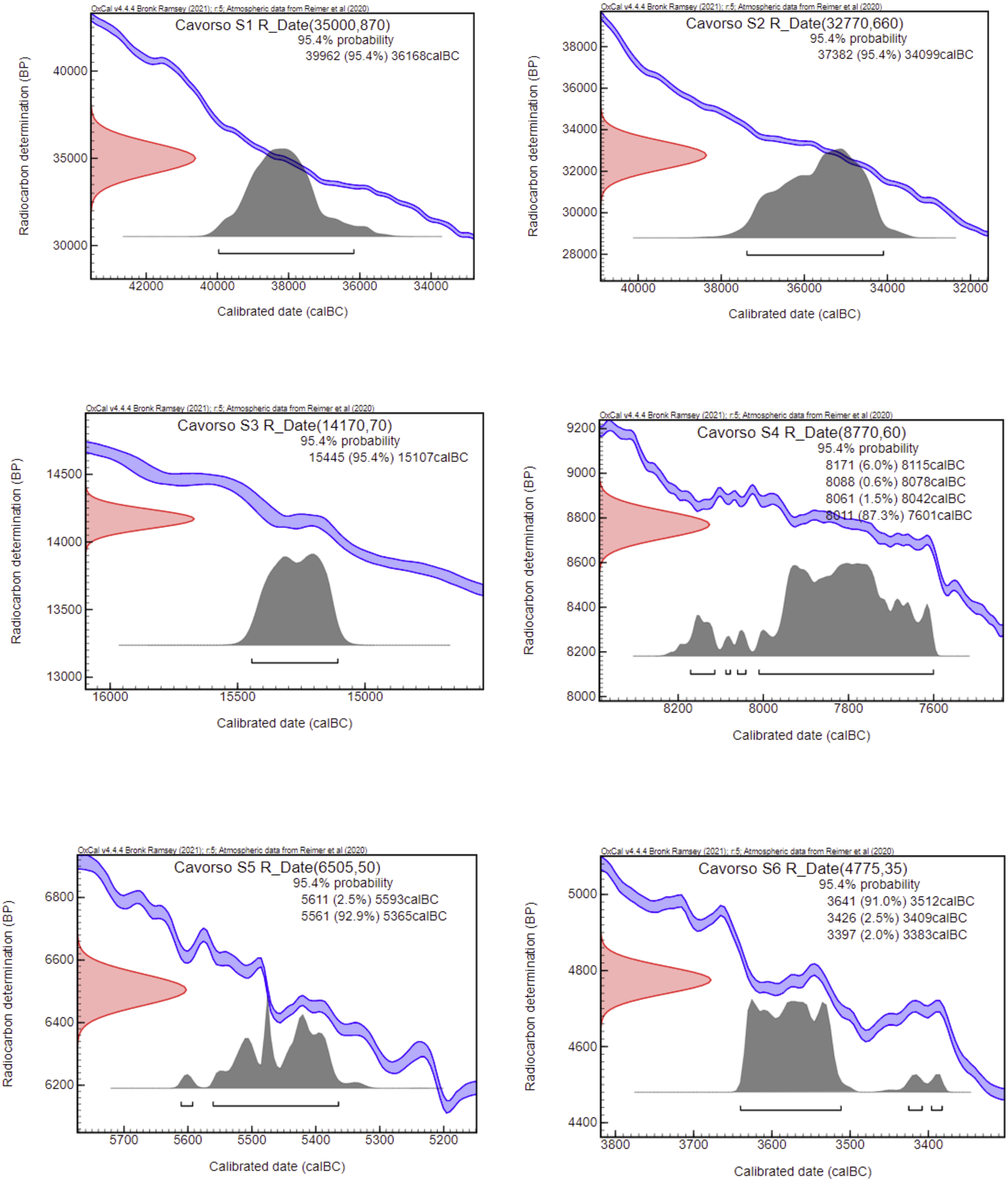

Supplement: Supplementary file 1 — Appendix S1 [file ECE3-12-e9486-s002.pdf]
