# Supplementary material for: Pollen record of the Late Pleistocene–Holocene stratigraphic sequence and current plant biodiversity from Grotta Mora Cavorso (Simbruini Mountains, Central Italy)
Source: Ecol Evol. 2022 Nov 8;12(11):e9486. doi: 10.1002/ece3.9486 (PMC9643123; doi:10.1002/ece3.9486)
Supplement: Supplementary file 2 — Appendix S2 [file ECE3-12-e9486-s001.pdf]

Pollen record of the Late Pleistocene-Holocene stratigraphic sequence and current plant biodiversity from Grotta Mora Cavorso (Simbruini mountains, Central Italy)

Alessia D’Agostino, Gabriele Di Marco, Silvia Marvelli, Marco Marchesini, Juan Manuel Martínez Labarga, Mario Federico Rolfo, Antonella Canini, Angelo Gismondi

SUPPLEMENTARY MATERIAL 2. Pollen percentage spectra of *Grotta Mora Cavorso* stratigraphic sequence.

GROTTA MORA CAVORSO

Jenne (Rome, Central Italy)

Pollen percentage spectra

SAMPLES (N°)			S1	S2	S3	S4	S5	S6	M
WOODY TAXA		GOUPS							
BETULACEAE	*Alnus* undiff.	T,DL,Hygr			3.7		1.9	6.9	1.9
	Carpinus betulus L.	T,DL,Q					1.0	3.9	10.4
	Corylus avellana L.	sh,DL,Q,EF	22.2		5.5		7.6	3.9	9.9
	Ostrya carpinifolia /*C. orientalis*	T,DL,Q	16.6	10.6	1.8	8.1	7.6	6.9	24.8
CISTACEAE	*Helianthemum*	sh,EV		5.3					
CORNACEAE	*Cornus mas* L.	T,DL,Q,EF						1.0	0.9
EPHEDRACEAE	*Ephedra fragilis* type	sh,EV						3.0	
FAGACEAE	*Castanea sativa* Miller	T,DL,EF					1.0	2.0	0.9
	Fagus sylvatica L.	T,DL,EF					1.0	1.0	
	Quercus ilex L.	T,EV,M,EF					1.0		4.3
	Quercus cf. *robur*	T,DL,Q,EF				1.4			2.8
	Quercus caducif. undiff.	T,DL,Q,EF				8.1	5.7	3.9	10.4
MALVACEAE	*Tilia cordata* Miller	T,DL,Q				1.4			
OLEACEAE	*Fraxinus excelsior* type	T,DL,Q	0.9						7.1
	Fraxinus ornus L.	T,DL,Q				2.7			3.8
	Fraxinus undiff.	T,DL,Q			11.1				9.6
PINACEAE	*Pinus* undiff.	T,EV,Cf	12.7	3.5	1.8	2.7	1.0	1.0	0.9
RHAMNACEAE	*Rhamnus* type	sh,DL,Q						1.0	0.5
SALICACEAE	*Salix*	T,DL,Hygr		1.8	5.5			5.9	0.9
ULMACEAE	*Ulmus*	T,DL,Q					1.0	2.0	
HERBACEOUS TAXA									
AMARANTHACEAE	*Chenopodium* cf.	As			0.9				
	Chenopodiaceae undiff.	As		2.7	2.8	1.4	2.9	3.0	0.5
APIACEAE	Apiaceae undiff.			0.9		1.4		1.0	0.9
ASTERACEAE	*Aster type*		0.9				1.9	3.9	
	Bellis perennis type	As				2.7			
	Centaurea nigra type	As	0.9	3.5	1.8	4.1		1.0	
	Asteroideae undiff.	pm		11.9	14.7	10.8	1.9	2.0	
	Cichorium intybus type	As,pm				1.4	10.5	12.8	
	Cichorioideae undiff.	pm	f.p	f.p.	f.p.	f.p.	f.p.	f.p.	f.p.
BORAGINACEAE	*Symphytum officinale* type						1.0		
BRASSICACEAE	*Hornungia* type				0.9		1.0	1.0	1.9
	Sinapis type				0.9				1.4
	Brassicaceae undiff.		0.9	7.1	5.1		1.0	6.9	2.4
CARYOPHYLLACEAE	Caryophyllaceae undiff.		0.9		0.9				
CYPERACEAE	Cyperaceae undiff.	hygro			0.9			1.0	
FABACEAE	*Hedysarum* cf.	As,pm						1.0	
	Lotus type				0.9		1.9	3.0	
	Fabaceae undiff.	pm	0.9			1.4	1.9	2.0	
LAMIACEAE	*Lamium amplexicaule* type	As			1.8		1.0		
	Salvia type						1.9		
	Stachys sylvatica type				0.9				
	Lamiaceae undiff.			2.7	2.8		1.0	1.0	0.9
POACEAE	"*Hordeum* " group	e,ce			0.9			1.0	
	Poaceae spontaneous group	pp	38.3	43.8	31.3	50.0	29.5	11.3	0.9
POLYGONACEAE	*Polygonum aviculare* group	As					1.0		
RANUNCULACEAE	*Ranunculus acris* type						1.9	1.0	0.9
	Ranunculaceae undiff.					1.4	3.8	1.0	0.5
ROSACEAE	Rosaceae undiff.						2.9	1.0	
RUBIACEAE	*Galium* type			0.9					
SCROPHULARIACEAE	Scrophulariaceae undiff.			0.9					
TYPHACEAE	*Typha angustifolia* L.	helo					1.0		
URTICACEAE	*Urtica dioica* type	As	2.8	0.9				1.0	
UNDETERMINATED GRAINS			1.8	3.5	2.8	1.4	3.8	2.0	0.5
INDETERMINABLE GRAINS			0.2	0.2	0.2	0.7	0.2	0.2	0.1
CICHOROIOIDEAE			61.2	66.0	65.0	89.8	55.8	56.5	
MONILOPHYTA									
THELYPTERIDACEAE	*Thelypteris*							7.9	
FILICALES MONOLETE			0.7	0.7		2.0	2.8	5.1	0.5
FILICALES TRILETE			1.8	0.4		0.7	0.9		
MONILOPHYTA-TOTAL			2.5	1.1		2.7	3.7	13.1	0.5
ALIA									
CONCENTRICYSTES							0.2		
SPORES OF MUSHROOMS			+		+		+	+	+
SECONDARY GRAINS									
	Cichorioideae					1.0	0.2		
	INDETERMINABLE		1.1		4.4	3.3	2.3	0.2	
SECONDARY GRAINS - TOTAL			1.1		4.4	4.2	2.6	0.2	
SUMMARY									
WOODY TAXA		T+sh+L	52.4	21.2	29.5	24.3	28.6	42.4	89.1
TREES		T	30.3	15.9	24.0	24.3	21.0	34.5	78.7
SHRUBS		sh	22.2	5.3	5.5		7.6	7.9	10.4
CONIFERS		Cf	12.7	3.5	1.8	2.7	1.0	1.0	0.9
EVERGREEN		EV	12.7	8.8	1.8	2.7	1.9	3.9	5.2
DECIDUOUS LEAVES		DL	39.7	12.4	27.6	21.6	26.7	38.4	83.9
TAXA QUERCETUM (*A. campestre type, C. betulus, O. carpinifolia/C. orientalis, Q. deciduous, Fraxinus, Tilia, Ulmus*)		Q(Ac+Cb+O/C+F+Qd+T+U)	17.6	10.6	12.9	21.6	15.2	18.7	70.2
QUERCETUM (T+sh)		Q = T+sh	39.7	10.6	18.4	21.6	22.9	22.7	80.2
WOODY HYGROPHYTES		Hygr		1.8	9.2		1.9	12.8	2.8
EDIBLE FRUITS WOODY		EF	22.2		5.5	9.5	16.2	11.8	29.3
MEDITERRANEAN TAXA		M					1.0		4.3
HERBACEOUS TAXA		H	47.6	78.8	70.5	75.7	71.4	57.6	10.9
HERBACEOUS HYGROPHYTES		hygro			0.9			1.0	
HELOPHYTES		helo					1.0		
HERBACEOUS HYGROPHYTES+HELOPHYTES		hygro+helo			0.9		1.0	1.0	
HERBACEOUS EDIBLE FRUITS		ef			0.9			1.0	
CEREALS		ce			0.9			1.0	
PASTURE-MEADOW		pm	39.3	55.8	46.1	63.5	43.8	29.1	0.9
HERBACEOUS ANTHROPIC SPONTANEOUS INDICATORS		As	3.7	7.1	7.4	9.5	15.2	18.7	0.5
TOTAL HYGROPHYTES-HYDROPHYTES+HELOPHYTES		Hygr+hygro+helo		1.8	10.1		2.9	13.8	2.8
POLLEN COUNTS	TOTAL								
TRACHEOPHYTA	4.015	S+M	523	527	538	515	519	542	851
SPERMATOPHYTA	3.949	S(T+sh+H)	512	522	538	507	503	520	847
MONILOPHYTA	66	M	11	5		8	16	22	4
SECONDARY GRANULES	41		5		20	10	10	1	
NUMBER OF TAXA TRACHEOPHYTA	TOTAL	T+sh+H+M							
	56		15	18	23	19	32	36	26
INDEX									
Floristic Richness Index		FRI	26.8	32.1	41.1	33.9	57.1	64.3	46.4
Human Influence on Flora Index		HIFI	7.0	33.3	25.0	38.9	53.3	44.2	0.5
CONCENTRATION (pollen grains/gram)									
TRACHEOPHYTA			3.232	8.872	74.637	12.596	26.841	26.917	1.442.410
SPERMATOPHYTA			3.164	8.788	74.637	12.401	26.013	25.825	1.435.631
MONILOPHYTA			68	84		196	827	1.093	6.780
SECONDARY GRANULES			31		2.775	245	517	50	
