# Supplementary material for: Pollen record of the Late Pleistocene–Holocene stratigraphic sequence and current plant biodiversity from Grotta Mora Cavorso (Simbruini Mountains, Central Italy)
Source: Ecol Evol. 2022 Nov 8;12(11):e9486. doi: 10.1002/ece3.9486 (PMC9643123; doi:10.1002/ece3.9486)
Supplement: Supplementary file 3 — Appendix S3 [file ECE3-12-e9486-s003.pdf]

**Pollen record of the Late Pleistocene-Holocene stratigraphic sequence and current plant biodiversity from Grotta Mora Cavorso (Simbruini mountains, Central Italy)**

Alessia D'Agostino, Gabriele Di Marco, Silvia Marvelli, Marco Marchesini, Juan Manuel Martínez Labarga, Mario Federico Rolfo, Antonella Canini, Angelo Gismondi

## **SUPPLEMENTARY MATERIAL 3.** Plant species identified by floristic *census*.

**FERNS AND FERN ALLIES**

ASPLENIACEAE

*Asplenium ceterach* L. subsp. *ceterach*

*Asplenium* cf. *onopteris* L.

*Asplenium trichomanes* L. subsp. *quadrivalens* D.E.Mey.

**ANGIOSPERMS**

**Monocotyledons**

ASPARAGACEAE

*Anthericum liliago* L.

*Asparagus acutifolius* L.

*Muscari comosum* (L.) Mill.

*Ruscus aculeatus* L.

CYPERACEAE

*Carex* cf. *divulsa* Stokes

ORCHIDACEAE

*Anacamptis pyramidalis* (L.) Rich.

POACEAE

*Achnatherum bromoides* (L.) P.Beauv. (=*Stipa bromoides* L.)

*Anisantha diandra* (Roth) Tutin ex Tzvelev (= *Bromus diandrus* Roth)

*Avena* sp.

*Brachypodium rupestre* (Host) Roem. & Schult.

*Bromopsis erecta* (Huds.) Fourr. subsp. *erecta* (= *Bromus erectus Huds.*)

*Calamagrostis* sp.

*Catapodium rigidum* (L.) C.E.Hubb. subsp. *rigidum*

*Cynosurus echinatus* L.

*Dactylis glomerata* L. subsp. *glomerata*

*Koeleria cf macrantha (Ledeb.) Schult. subsp. macrantha*

*Lolium rigidum* Gaudin

*Phleum* cf. *hirsutum* Honck. subsp. *ambiguum* (Ten.) Cif. & Giacom.

**Dicotyledons**

ANACARDIACEAE

*Pistacia terebinthus* L. subsp. *terebinthus*

APIACEAE

*Bupleurum baldense* Turra

*Eryngium amethystinum* L.

*Tordylium apulum* L.

*Tordylium maximum* L.

*Torilis africana* Spreng.

*Torilis* cf. *elongata* (Hoffmanns. & Link) Samp.

ARISTOLOCHIACEAE

*Aristolochia lutea Desf.*

ASTERACEAE

*Anthemis* sp.

*Bellis* cf. *annua* L.

*Carduus* sp.

*Crepis* cf. *capillaris* (L.) Wallr.

*Crepis* cf. *neglecta* L. subsp. *neglecta*

*Erigeron* cf. *sumatrensis* Retz.

*Eupatorium cannabinum* L. subsp. *cannabinum*

*Helichrysum italicum* (Roth) G.Don subsp. *italicum*

*Hieracium* sp.

*Inula* sp.

*Lactuca viminea* (L.) J.Presl & C.Presl subsp. *chondrilliflora* (Boreau) St.-Lag.

*Phagnalon sordidum* (L.) Rchb.

*Picris* sp.

*Reichardia picroides* (L.) Roth

BETULACEAE

*Corylus avellana* L.

*Ostrya carpinifolia* Scop.

BORAGINACEAE

*Echium italicum* L. subsp. *italicum*

BRASSICACEAE

*Arabis* cf. *collina* Ten

*Erysimum apenninum* Peccenini & Polatschek

*Hesperis laciniata* All. subsp. *laciniata*

*Pseudoturritis turrita* (L.) Al-Shehbaz (=*Arabis turrita* L.)

*Thlaspi* cf. *alliaceum* L.

<u>CAMPANULACEAE</u>

*Campanula glomerata* L.

*Campanula rapunculus* L.

<u>CAPRIFOLIACEAE</u>

*Lonicera etrusca* Santi

<u>CARYOPHYLLACEAE</u>

*Gypsophila repens* L.

*Silene* sp.

*Silene saxifraga* L.

*Silene vulgaris* (Moench) Garcke subsp. *vulgaris*

<u>CISTACEAE</u>

*Helianthemum apenninum* (L.) Mill. subsp. *apenninum*

*Helianthemum nummularium* (L.) Mill.

*Helianthemum oelandicum* (L.) Dum.Cours. subsp. *italicum* (L.) Ces.

<u>CONVOLVULACEAE</u>

*Convolvulus cantabrica* L.

<u>CORNACEAE</u>

*Cornus sanguinea* L. subsp. *sanguinea*

<u>CRASSULACEAE</u>

*Petrosedum* cf. *montanum* (Songeon & E.P.Perrier) Grulich

*Sedum album* L. subsp. *album*

*Sedum sexangulare* L.

<u>DIOSCOREACEAE</u>

*Dioscorea communis* (L.) Caddick & Wilkin (= *Tamus communis* L.)

<u>DIPSACACEAE</u>

*Sixalix atropurpurea* (L.) Greuter & Burdet (= *Scabiosa atropurpurea* L.)

<u>FABACEAE</u>

*Argyrolobium zanonii* (Turra) P.W.Ball subsp. *zanonii*

*Astragalus* cf. *hamosus* L.

*Colutea arborescens* L.

*Cytisophyllum sessilifolium* (L.) O.Lang

*Cytisus villosus* Pourr.

*Emerus major* Mill. subsp. *major*

*Ervilia* cf. *loiseleurii* (M.Bieb.) H.Schaef.

*Genista monspessulana* (L.) L.A.S.Johnson (= *Teline monspessulana* L.)

*Hippocrepis comosa* L. subsp. *comosa*

*Lathyrus aphaca* L. subsp. *aphaca*

*Medicago arabica* (L.) Huds.

*Medicago lupulina* L.

*Trifolium campestre* Schreb.

*Trifolium ochroleucon* Huds.

*Trifolium pratense* L. subsp. *pratense*

*Trifolium repens* L.

*Trifolium stellatum* L.

*Vicia cf dumetorum* L.

<u>FAGACEAE</u>

*Quercus ilex* L. subsp. *ilex*

*Quercus pubescens* Willd. subsp. *pubescens*

<u>GERANIACEAE</u>

*Geranium* sp.

*Geranium purpureum* Vill.

<u>HYPERICACEAE (= GUTTIFERAE)</u>

*Hypericum perforatum* L.

<u>LAMIACEAE</u>

*Clinopodium alpinum* (L.) Kuntze (=*Acinos alpinus* L.)

*Glechoma* sp.

*Melittis melissophyllum* L. subsp. *melissophyllum*

*Micromeria gr graeca* (L.) Benth. ex Rchb.

*Prunella laciniata* (L.) L.

*Satureja montana* L. subsp. *montana*

*Stachys germanica* L. subsp. *salviifolia* (Ten.) Gams

*Stachys sylvatica* L.

*Teucrium chamaedrys* L. subsp. *chamaedrys*

<u>MALVACEAE</u>

*Malva* sp.

<u>MORACEAE</u>

*Ficus carica* L.

<u>OLEACEAE</u>

*Fraxinus ornus* L. subsp. *ornus*

<u>PAPAVERACEAE</u>

*Chelidonium majus* L.

<u>POLYGONACEAE</u>

*Rumex* cf. *obtusifolius* L. subsp. *obtusifolius*

<u>PLANTAGINACEAE</u>

*Plantago lanceolata* L.

*Plantago major* L.

<u>RANUNCULACEAE</u>

*Clematis vitalba* L.

*Helleborus foetidus* L. subsp. *foetidus*

*Ranunculus* sp.

<u>ROSACEAE</u>

*Crataegus monogyna* Jacq.

*Fragaria vesca* L. subsp. *vesca*

*Poterium sanguisorba* L. subsp. *balearicum* (Bourg. ex Nyman) Stace (= *Sanguisorba minor* subsp. *balearica*)

*Potentilla* cf. *recta*

*Prunus mahaleb* L. subsp. *mahaleb*

*Rosa* sp.

*Rubus ulmifolius* Schott

<u>RUBIACEAE</u>

*Asperula* cf. *aristata* L. f. subsp. *aristata*

*Cruciata glabra* (L.) C. Bauhin ex Opiz

*Cruciata laevipes* Opiz

*Galium* sp.

*Galium aparine* L.

*Galium odoratum* (L.) Scop.

*Sherardia arvensis* L.

<u>SAPINDACEAE</u>

*Acer campestre* L.

*Acer monspessulanum* L. subsp. *monspessulanum*

*Acer opalus* Mill. subsp. *obtusatum* (Waldst. & Kit. ex Willd.) Gams

<u>SCROPHULARIACEAE</u>

*Scrophularia canina* L.

*Verbascum macrurum* Ten.

<u>URTICACEAE</u>

*Parietaria judaica* L.

*Urtica dioica* L. *subsp. dioica*

<u>VALERIANACEAE</u>

*Valeriana* sp.

<u>VIBURNACEAE</u>

*Sambucus ebulus* L.

VIOLACEAE

*Viola* sp.

*Viola* cf. *odorata* L.
